# Celecoxib-Loaded Electrospun Fibrous Antiadhesion Membranes Reduce COX-2/PGE_2_ Induced Inflammation and Epidural Fibrosis in a Rat Failed Back Surgery Syndrome Model

**DOI:** 10.1155/2021/6684176

**Published:** 2021-02-23

**Authors:** Wei Wang, Yunhao Wang, Tengfei Lou, Mingqian Ding, Juehong Li, Hao Xiong, Zhixiao Yao, Yingying Ma, Huajiang Chen, Shenghe Liu

**Affiliations:** ^1^Department of Orthopaedics, Shanghai Jiao Tong University Affiliated Sixth People's Hospital, 600 Yishan Road, Shanghai, China; ^2^Department of Spinal Surgery, Changzheng Hospital Affiliated to Second Military Medical University, 415 Fengyang Road, Shanghai 200003, China; ^3^Taian TSCM Hospital, No. 265 Lingshan Street, Taian, Shandong 271000, China; ^4^Department of Medical Engineering, Shandong Provincial Hospital Affiliated to Shandong University, 423 5th Longitude Crossing 7th Latitude Road, Shandong 250021, China

## Abstract

To date, failed back surgery syndrome (FBSS) remains a therapy-refractory clinical condition after spinal surgery. The antiadhesion membrane is applied to prevent FBSS by isolating fibrosis; however, the inflammation stimulated by the foreign body and surgical trauma needs to be further resolved simultaneously. Therefore, we developed new electrospun polycaprolactone (PCL) fibrous membranes loaded with celecoxib (CEL) to prevent fibrosis and inflammation associated with FBSS. The CEL-loaded PCL fibers were randomly distributed, and the drug was released over two weeks. Fluorescence micrographs revealed that the fibroblasts proliferated less on the PCL-CEL fibrous membranes than in the PCL group and the blank control. In the rat laminectomy model after 4 weeks, magnetic resonance imaging of epidural fibrosis was least in the PCL-CEL group. Expression of COX-2 and PGE_2_ was lower in the PCL-CEL group. It concluded that the CEL-loaded PCL membrane could reduce fibrosis and inflammation in a rat model of FBSS via COX-2/PGE_2_ signaling pathways.

## 1. Introduction

Until now, failed back surgery syndrome (FBSS) remains a therapy-refractory clinical complication after spinal surgery [1–3]. A characteristic manifestation of FBSS is persistent low back pain, leading to disability, low quality of life, and unemployment [4, 5]. It is estimated to occur in 13–61% of all postoperative patients [6, 7]. Considering the increased incidence of spinal surgeries, the high associated costs, and the increasing prevalence of FBSS, the cost burden of this disease is a significant problem [3].

Epidural fibrosis caused by migrated paravertebral fibroblasts is generally accepted as the pathologic mechanism of FBSS. However, the degree of compression of the epidural scar revealed by imaging analysis shows an ambiguous relationship with the severity of clinical symptoms of FBSS [8, 9]. Inspired by clinical findings, scientists found that inflammation-related arachnoiditis was a potential pathologic mechanism of FBSS rather than mechanical compression [10, 11]. Although an antifibrotic membrane was used to prevent FBSS by isolating the immigration of fibrotic factors [12, 13], the inflammation stimulated by foreign bodies and biodegradation products [14, 15] overwhelms the advantage of a physical barrier. To date, the pathophysiological mechanism in the development of FBSS is under investigation.

It is well known that inflammation is involved in arachidonic acid metabolism, in which cyclooxygenase-2 (COX-2) and prostaglandin E2 (PGE_2_) signaling plays key roles in the migration and proliferation of fibroblasts [16–18]. Meanwhile, a selected cyclooxygenase-2 inhibitor (celecoxib (CEL)) was found to downregulate ERK1/2 and SMAD2/3 phosphorylation, leading to reduced collagen I and collagen III expression, inflammatory reactions, and fibroblast proliferation [19]. A recent study found that macrophages and COX-2 can be triggered by fibrous membranes and may lead to subsequent inflammation and granuloma formation [14, 15]. Thus, the downregulation of COX-2 expression might reduce the adhesion induced by trauma (surgery) and foreign bodies in the pathophysiological processes of FBSS.

PGE_2_ is a crucial mediator of inflammatory pain sensitization [20, 21]. Furthermore, prostaglandin-mediated modulation of serotonergic transmission controls the affective component of inflammatory pain [22], making PGE_2_ more potent in the pain mechanism of inflammation. Furthermore, a study has shown that PGE_2_ may facilitate the transmission of nociceptive input through the spinal cord dorsal horn to higher brain areas where pain becomes conscious [23]. These findings suggest that PGE_2_ could play an essential role in the pain mechanism of inflammation-related arachnoiditis in FBSS. However, few studies have revealed the relationship between PGE and FBSS.

Selective nonsteroidal anti-inflammatory agents (such as CEL) are highly selective against COX-2. They are widely used in clinics with acceptable safety profiles [24]. Consequently, selective nonsteroidal anti-inflammatory agents would be an ideal drug for reducing adhesion formation and inflammation to prevent FBSS induced by fibrous biomaterials. In this study, we developed new electrospun polycaprolactone (PCL) fibrous membranes loaded with CEL. To investigate its antiadhesion, anti-inflammation, and pain relief functions via the COX-2/PGE_2_ signaling pathways, *in vitro* cell experiments and rat laminectomy models were performed.

## 2. Materials and Methods

### 2.1. Preparation of CEL-Loaded Electrospun Fibrous Membranes

PCL (Mw = 80 kDa) and CEL were analytical reagents acquired from Sigma-Aldrich (Saint Louis, MO, USA). The electrospun techniques were performed according to our previous study [12]. Briefly, PCL-CEL fibrous membranes (CEL-loaded PCL) were produced by completely dissolving 1 g of PCL and 20 mg (0.2% wt/v) or 60 mg CEL (0.6% wt/v) in 10 mL 1,1,1,3,3,3-hexafluoro-2-propanol (HFIP, Shanghai Darui Fine Chemical Co., Ltd.). The solution was then fed at 2 mL/h by a syringe pump, electrospun at 15 kV, and collected by a slowly rotating mandrel. The obtained fibrous membranes were dried at room temperature under vacuum for at least one week to remove residual solvent.

### 2.2. SEM Characterizations of PCL Electrospun Membranes

Scanning electron microscopy (SEM, FEI Quanta 200, Netherlands) was used to detect the morphological characteristics of the PCL electrospun membranes loaded or unloaded with CEL. At least five images of each sample were investigated (10,000x magnification).

### 2.3. CEL Release Characteristics of Fibrous Membranes

Exactly 100 mg of each fibrous membrane sample was immersed in 20 mL of phosphate-buffered saline (PBS, pH 7.4) with lipase (0.05 mg/mL). The release kinetics of CEL were investigated in a thermostatic shaking water bath (37°C, Taichang Medical Apparatus Co., Jiangsu, China) at a frequency of 100 cycles per minute. Equal portions of the release buffer (5.0 mL each) were collected and refreshed with 5.0 mL PBS at scheduled time points. The release of CEL was determined by spectrophotometry (Shimadzu, Japan) at UV-2550.

### 2.4. *In Vitro* Cell Culture

Chicken embryonic fibroblasts (UMNSAH/DF-1) were used to evaluate the adhesion and proliferation on the surfaces of CEL-loaded and non-CEL-loaded fibrous membranes. The cells were cultured in Dulbecco's modified Eagle's medium (supplemented with 10% fetal bovine serum, 100 U/mL penicillin, and 100 mg/mL streptomycin) at 37°C with 5% CO_2_ atmosphere with culture medium replaced three times per week. For timely collection, cells were digested with 0.25% trypsin and washed with PBS for 90 min. Finally, a 24-well plate held 100 mL per well of transferred samples with a density of 1 × 10^5^ cells/mL.

### 2.5. Cell Proliferation and Viability Assay

100 *μ*L of the cell suspension (1 × 10^5^ cells/mL) was seeded into each well of 96-well plates, and the plates were incubated for 1, 3, or 5 days (37°C, 5% CO_2_) to allow the cells to adhere to the wall. At each time point, cells were incubated in the medium at 10% volume (10 *μ*L) mixed with Cell Counting Kit-8 (CCK-8, Dojindo, Japan). The absorbance was measured at 450 nm with a microplate reader (Multiskan Mk3, Thermo Fisher Scientific, USA). The cell number was calculated based on the CCK-8 standard curve.

The influence of the surface of different materials on cell viability was assessed using a live/dead staining kit (Invitrogen, Eugene, OR). In the 24-well plates, cells were cultured in each well with a density of 4 × 10^4^/cm^2^ for 24 h. The cells were collected, washed twice with PBS, and then resuspended in 2 mM calcein-AM and 10 mM EthD-1 staining solution, then incubated at 4°C in the dark for 30 min. Finally, under a confocal laser scanning microscope (Leica TCS SP2, Heidelberg, Germany), the stained cells were observed. The membranes of living cells were stained bright green, while the dead cell membranes were red; thus, the dead/live cell ratio was obtained. All experiments were repeated three times.

### 2.6. Animal Model

The ethics and safety of the animal experiments were ensured following the guidelines of Shanghai Jiao Tong University. Male Sprague-Dawley rats (provided by Shanghai SLAC Laboratory Co., Ltd.) (300 ± 20 g) were divided into three groups. Electrospun fibrous membranes were prepared with dimensions of 4 mm × 6 mm. Anesthesia was performed using intramuscular ketamine injected at a dose of 20 mg/kg. After sterilization, the second through fifth lumbar vertebral spinous processes were excised under surgical microscopy through the dorsal approach [25, 26]. The dura mater was protected while exposing the spinal cord. The blank group (without membranes), the control group (non-CEL-loaded membranes), and the experimental groups (CEL-loaded membranes) were defined after placing fibrous membranes on the dura mater. Finally, the incision was closed. The exclusion criteria of animal models included dura mater lesions during surgery, postoperative infection, and neurological defects. The procedures were performed by trained surgeons. Rats were sacrificed with a lethal dose of anesthetic after 4 weeks of feeding. Before intact vertebral columns were harvested for histological and molecular biological assays, animals underwent magnetic resonance imaging to evaluate epidural fibrosis.

### 2.7. MRI Assessment of Epidural Fibrosis

After 4 weeks, the animals underwent magnetic resonance imaging (MRI) analysis to investigate epidural fibrosis. All animals were imaged on a Siemens Magnetom Verio 3.0 T MRI system (Siemens, Germany), with a 19-layer cross-section of the spine. Following localization images, the TRA MRI sequence was used (repetition time/time to echo 4970/114 ms, with a field-of-view of 66 × 96 mm, and a 282 × 512 matrix). The range of epidural fibrosis was evaluated according to the following criteria: Grade 0, no epidural fibrosis; Grade 1, only thin adhesive bands were observed on the dura and scar tissue; Grade 2, epidural fibrosis observed in <2/3 of the laminectomy site; and Grade 3, large scar tissue affecting >2/3 of the laminectomy site, or the adherence extends to the nerve roots [27, 28]. Two investigators who were blinded to the treatment performed this evaluation independently.

### 2.8. Inflammation Assessment

The expression of COX-2 and PGE_2_ was investigated using immunohistochemical staining to detect inflammation in epidural fibrosis tissue. First, the dewaxed and dehydrated parts of xylene were carried out in a continuous gradient concentration of ethanol. Then, they were treated in methanol with 3% hydrogen peroxide for 10 min. To extract the antigen, histologic sections were soaked in 100°C 10 mM citrate buffer (pH = 6.0) for 10 min, then diluted in PBS (pH = 7.4) to 1 : 200 of COX-2 (Abcam) and PGE_2_ (Abcam) primary antibodies for incubation, and stored overnight at 4°C. The samples were incubated with rabbit anti-COX-2 and anti-PGE_2_ antibodies at 37°C for 1 h after washing with PBS. The peroxide reaction was performed as a chromogenic agent, and DAPI was used to counterstain the slides. To quantify, we investigated COX-2 and PGE_2_ in a physical barrier plantation. The tissue area normalization of each sample (*n* = 10) was used to detect the degree of inflammation in each area.

### 2.9. Statistical Analysis

In general, the results are presented as the means ± standard deviation (SD) values. One-way analysis of variance (ANOVA) was used to analyze the data among each group. A *P* value < 0.05 was considered statistically significant. SPSS 10.0 (Chicago, IL, USA) was used for the data analysis of this study.

## 3. Results

### 3.1. SEM Appearance and Drug Release Characteristics of Fibrous Membranes

**Figure 1:**
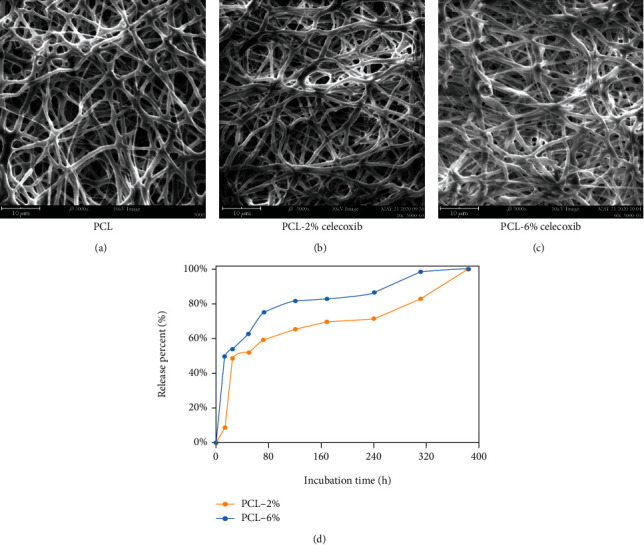
SEM observation of PCL fibrous membranes (a), PCL-2%-CEL fibrous membrane (b), and PCL-6%-CEL fibrous membrane (c). The cumulative release profiles of CEL-loaded PCL fibrous membranes (d).

**Figure 2:**
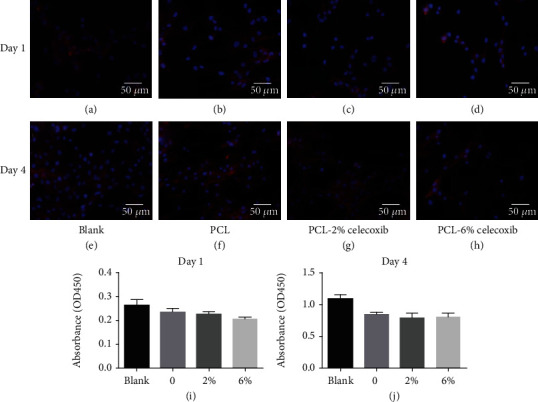
Fluorescence micrographs of chicken embryonic fibroblasts (UMNSAH/DF-1) after 24 h and 4 days of incubation. The cytoskeleton was stained red, and the nuclei were stained blue on the surfaces of the fibrous membranes. The fibroblast proliferation on the sample surfaces (*n* = 3), blank (a, e), PCL fibrous membranes (b, f), PCL-20%-CEL fibrous membranes (c, g), and PCL-60%-CEL fibrous membranes (d, h).

SEM images of electrospun fibrous membranes (Figures 1(a)–1(c)) showed that the fibers were randomly distributed in a well-distributed manner. All the microfibers were round, continuous, and bead-free. The surface of the CEL-loaded fibers was smooth with no drug crystallization. The surface structures were not significantly different among 2%- and 6%-CEL-loaded fibers and control fibers. The porosity of CEL-loaded PCL fibrous scaffolds was 58.9%, 61.2%, and 63.8% for PCL, PCL-2%, and PCL-6%, respectively, and the porosity was not significantly affected by the drug incorporation. The release profiles of CEL are shown in Figure 2. It took nearly one week (2% CEL) and two weeks (6% CEL) for the PCL-CEL electrospun fibrous membrane to release approximately 80% of the loaded drug with a burst release of about 50% of the drug in the first 2 days (Figure 1(d)). Meanwhile, the PCL-2%-CEL electrospun fibrous membrane presented a longer release duration than that of the PCL-6%-CEL.

### 3.2. Cell Proliferation and Viability

Figure 2 shows fibroblast proliferation on the PCL-CEL and PCL-control fibrous membranes one and four days after culture. Fluorescence micrographs showed that the adherence of fibroblasts was less on the PCL-CEL fibrous membranes than on the PCL membranes and in the blank controls (Figures 2(a)–2(j)).

### 3.3. *In Vivo* Magnetic Resonance Imaging of the Extent of Fibrosis

**Figure 3:**
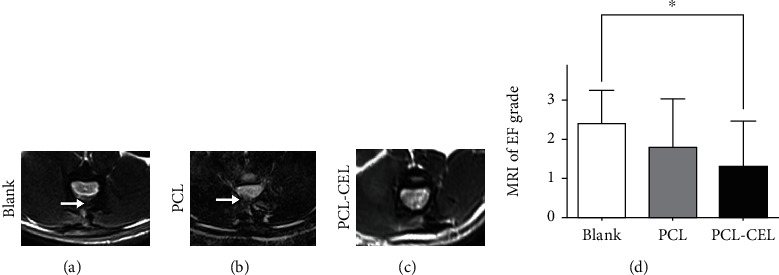
Magnetic resonance imaging (MRI) of the spine. MRI images of epidural fibrosis in the control group (a), the PCL group (b), and the PCL-CEL group. Epidural fibrosis grades based on MRI images (d). White arrows indicate epidural fibrosis. ^∗^*P* < 0.05. EF: epidural fibrosis.

Four weeks after laminectomies, dense epidural fibrosis tissues with widespread adhesions to the dura mater were detected by MRI examination at the caudal surgical sites in the control group (Figure 3(a)). More adhesion tissues were found in the laminectomy site of the PCL group than in that of the PCL-CEL group in the cross-sectional MRI images (Figure 3(b)), and few epidural adhesion tissues with well-defined dura were seen after 4 weeks in the PCL-CEL group (Figure 3(c)). According to the grading of extent of epidural fibrosis, the score of the control group was larger than that of the PCL-CEL group with significant difference at 4 weeks and also larger than that of the PCL group with significant difference (Figure 3(d)).

### 3.4. *In Vivo* Evaluation of Inflammation

**Figure 4:**
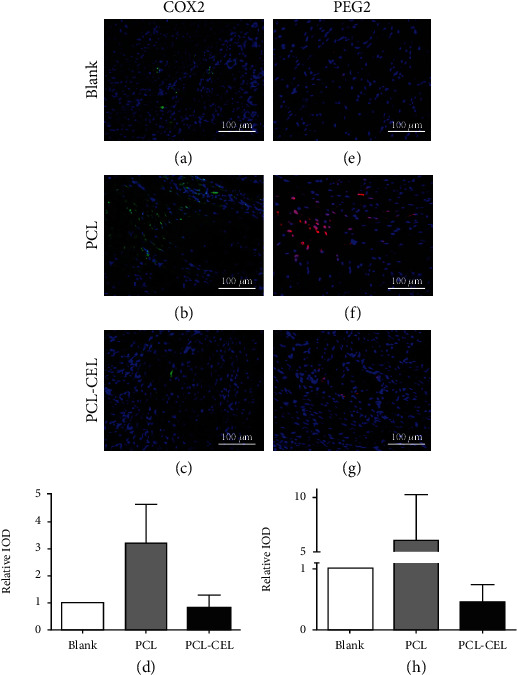
Immunofluorescence of tissue around epidural fibrosis. (a–d) Expression of COX-2; (e–h) expression of PGE_2_.

Immunofluorescence staining at 4 weeks showed that the amount of COX-2 and PGE_2_ in the PCL group was significantly higher than that in the PCL-CEL and control groups, whereas it was lowest in the PCL-CEL group (Figure 4).

## 4. Discussion

In this study, we investigated the effect of CEL-loaded PCL antiadhesion membranes in preventing FBSS, and its related mechanism. The drug release characteristics of this novel biomaterial ensured sufficient working time for surgical trauma and secondary inflammatory responses. *In vivo*, the density of epidural fibrosis was lower in the laminectomy site in the CEL-loaded PCL group compared to the control group based on the MRI assessment. Meanwhile, the nerve function of the CEL-loaded PCL group was significantly better than that of the control group. Furthermore, immunofluorescence staining indicated that the expression of COX-2 and PGE_2_ was lower in the CEL-loaded PCL group.

The pathophysiological bases of FBSS are epidural fibrosis and inflammation-related arachnoiditis [10, 11]. Studies have shown that the severity of clinical symptoms of FBSS could not be confirmed by imaging analysis of the epidural fibrosis clinically [8, 9]; however, in an endoscopic study in patients with persistent pain after extensive back surgery, 91.1% had severe epidural fibrosis, of which 84.3% of patients presented with concordant pain [29]. Many studies have revealed a positive correlation between inflammation and fibrosis or adhesion formation [25, 30, 31]. Overall, this suggests that inflammation is one of the most critical pathophysiological mechanisms in the development of FBSS.

Recent studies have found that the expression of COX-2 is upregulated in the fibrosis of diseases such as cirrhosis, chronic obstructive pulmonary disease, chronic kidney disease, cardiac hypertrophy, and tendon adhesion [32–36]. The extracellular signal-regulated kinase 2 and SMAD signaling pathways are the most likely mechanisms in COX-2-related fibrosis. Jiang et al. reported that a selective cyclooxygenase-2 inhibitor (CEL) inhibited ERK1/2 and SMAD2/3 phosphorylation, and as a result, the expression of collagen I/III and the proliferation of fibroblasts were reduced [19]. Meanwhile, it was found that the macrophage/COX-2 pathway can be triggered by antiadhesion fibrous membranes, leading to subsequent granuloma formation [14, 15]. Thus, downregulation of COX-2 expression might reduce the adhesion formation induced by trauma (surgery) and foreign bodies in the pathophysiological process of FBSS. In this study, the expression of COX-2 was significantly lower in the PCL-CEL group, and the extent of adhesion to the dura mater was lower in the MRI assessment.

It is well known that inflammation is based on arachidonic acid metabolism, in which the COX-2/PGE_2_ pathway plays a key role [16–18]. PGE_2_ is a crucial mediator of inflammatory pain sensitization [20–22], which can further facilitate the transmission of nociceptive input through the spinal cord dorsal horn to higher brain areas where pain reaches consciousness [23]. In addition, foreign bodies such as fibrous membranes could trigger macrophages and COX-2, which would lead to subsequent inflammation [14, 15]. In the three groups of this study, the expression of COX-2 and PGE_2_ at four weeks postoperatively was highest in the PCL group (higher than in the control group), whereas it was lowest in the PCL-CEL group. Considering the similar relative pain analysis trends in the nerve function assessment, these findings suggested that fibrous biomaterial served as an antiadhesion barrier that could trigger inflammation.

Meanwhile, the CEL-loaded fibrous PCL membranes reversed the COX-2-based inflammation and reduced pain by downregulating PGE_2_ expression. To our knowledge, this is the first study to reveal the role of PGE in the process of FBSS. Based on the crosslinking of adhesion and inflammation, selected cyclooxygenase-2 inhibitors could be ideal agents for preventing the epidural fibrosis and inflammation associated with FBSS. Further in-depth studies are required to understand the mechanism fully before clinical application. For example, there are four known PGE_2_ receptors: EP1, EP2, EP3, and EP4. Of these, EP4 expression in tenocytes was much higher than any other PGE_2_ receptor type [37]. The influence of CEL-loaded fibrous membranes on these receptors in surgical sites should be further investigated.

There are some shortcomings in this study. First, the observation time of the animal model was not very long. However, the levels of inflammation factors and extent of fibrosis were similar between 4 weeks and longer time points [26]. Second, a selective antagonist of PGE_2_ receptor 4 (EP4) was not detected, and further research should be performed to clarify the relevant mechanisms. Finally, the sample size needs to be expanded in subsequent studies.

## 5. Conclusions

In a rat laminectomy model, CEL-loaded PCL membranes could reduce fibrosis and inflammation associated with FBSS via the COX-2/PGE_2_ signaling pathways. Further evidence is needed for clinical application of these findings.

## Data Availability

All data used to support the findings of this study are available from the corresponding authors upon request.
